# Sleep-Enhancing Effect of Water Extract from Jujube (*Zizyphus jujuba* Mill.) Seeds Fermented by *Lactobacillus brevis* L32

**DOI:** 10.3390/foods12152864

**Published:** 2023-07-27

**Authors:** Gi Yeon Bae, Yejin Ahn, Ki-Bae Hong, Eun-Jin Jung, Hyung Joo Suh, Kyungae Jo

**Affiliations:** 1 Department of Integrated Biomedical and Life Science, Graduate School, Korea University, Seoul 02841, Republic of Korea; rldus530@naver.com (G.Y.B.); ahnyj708@gmail.com (Y.A.); 2 Department of Food Science and Nutrition, Jeju National University, Jeju 632943, Republic of Korea; kbhong@jejunu.ac.kr; 3 Department of Food and Biotechnology, Nutrition, Korea University, Sejong 30019, Republic of Korea; ejjung1124@korea.ac.kr; 4 Transdisciplinary Major in Learning Health Systems, Department of Healthcare Sciences, Graduate School, Korea University, Seoul 02841, Republic of Korea

**Keywords:** *Zizyphus jujuba*, enzymatic hydrolysis, fermentation, sleep, GABA_A_ receptor

## Abstract

Although *Ziziphus jujuba* Mill (jujube) is used in folk medicine for hypnotic sedative, anxiolytic, and many other purposes, to date, only a few studies have revealed its sleep-promoting effects and related mechanisms. Currently, drugs used for the treatment of sleep disorders have various side effects, so it is essential to develop safe natural materials. Therefore, we evaluated the sleep-enhancing activity and mechanism of action of an aqueous extract of jujube seeds (ZW) fermented with *Lactobacillus brevis* L-32 in rodent models. The starch contained in ZW was removed by enzymatic degradation and fermented with *L. brevis* to obtain a fermented product (ZW-FM) with a high γ-aminobutyric acid (GABA) content. To evaluate the sleep-promoting effect of ZW-FM, pentobarbital-induced sleep tests were performed on ICR mice, and electroencephalography analysis was undertaken in Sprague Dawley rats. Additionally, the awakening relief effects of ZW-FM were confirmed in a caffeine-induced insomnia model. Finally, the mechanism of sleep enhancement by ZW-FM was analyzed using GABA receptor type A (GABA_A_) antagonists. The ZW-FM-treated groups (100 and 150 mg/kg) showed increased sleep time, especially the δ-wave time during non-rapid eye movement (NREM) sleep. In addition, the 150 mg/kg ZW-FM treatment group showed decreased sleep latency and increased sleep time in the insomnia model. In particular, NREM sleep time was increased and REM sleep time, which was increased by caffeine treatment, was decreased by ZW-FM treatment. ZW-FM-induced sleep increase was inhibited by the GABA_A_ receptor antagonists picrotoxin, bicuculline, and flumazenil, confirming that the increase was the result of a GABAergic mechanism. These results strongly suggest that the increased GABA in water extract from jujube seeds fermented by *L. brevis* acts as a sleep-promoting compound and that the sleep-promoting activity is related to GABA_A_ receptor binding.

## 1. Introduction

Sleep is a state of rest and one of the most basic life phenomena. People in modern society suffer from acute and temporary sleep disorders in their complicated lives. Lack of sleep changes the normal circadian rhythm, resulting in various physical and mental symptoms, such as fatigue, helplessness, loss of motivation, endocrine disorders, cardiovascular disorders, cognitive decline, and mood decline [1,2]. Approximately 30–35% of the total population has temporary sleep disorders, especially women and the elderly. The drugs used currently for the treatment of sleep disorders include benzodiazepines (BDZ), non-BDZ medications, and antidepressants. These drugs often result in symptoms such as anxiety, agitation, and withdrawal, which can lead to difficulties in daily life as well as risk factors for cognitive impairment, depression, and other diseases [3]. Therefore, there is an urgent need to develop alternative foods and medications that can improve sleep disorders with fewer side effects [4].

Research is underway to develop sleep-inducing supplements that contain substances such as adenosine, acetylcholine, serotonin, melatonin, and gamma-aminobutyric acid (GABA) [5]. In addition, various herbal extracts, such as valerian (*Valeriana officinalis* L.), ashwagandha (*Withania somnifera* L.), lettuce (*Lactuca sativa* L.), hops (*Humulus lupulus* L.), jujube (*Ziziphus jujuba* Mill.), and chrysanthemum (*Chrysanthemum morifolium* Ramat.), have been developed as sleep-enhancing agents.

*Z. jujuba* (jujube), a member of the family Rhamnaceae, commonly known as Bor, is widely distributed in Southeast Asia, including Korea, and its seeds have been used for the treatment of insomnia since ancient times because of their calming effect on nerves [6]. Flavonoids, polysaccharides, and triterpenic acid present in jujube seeds have been reported as the major active ingredients [7,8,9], and alkaloid (sanjoinine A) and saponins (jujuboside A) have sedative and sleep-enhancing effects. Sanjoinine A and jujuboside enhance pentobarbital-induced sleep behavior in mice, which has been demonstrated by GABAergic system modulation [6,10]. However, jujube seeds were shown to have low contents of jujuboside (0.8 mg/g) [11] and pinosyn (1–0.4 mg/g) [12]; hence, it is highly likely that the seeds have other active ingredients. GABA has also been documented to be a sleep-enhancing ingredient in jujube seeds, with an amount of 333.37–150.31 μg/g being reported in six jujube varieties [13].

GABA is a neurotransmitter inhibitor and a non-protein amino acid present in the brain and spinal cord of bacteria, plants, and animals. GABA is effective in improving cerebral blood flow, relieving stress, enhancing memory, lowering blood pressure, relieving depression, and preventing dementia and insomnia [14]; the efficacy is similar to that of jujube seeds [13]. GABA also plays a central role in the treatment of sleep disorders. For the physiological activity of GABA, 50–200 mg per day needs to be consumed [15], but since the content of GABA present in natural products is minimal, research is being actively conducted to induce high concentrations of GABA [16]. As GABA is presumed to be a sleep-enhancing component of jujube seeds, in this study, fermentation using *L. brevis* L32 was performed to increase its content. This study aimed to evaluate the sleep-enhancing activity of jujube seeds, whose GABA content was increased by fermentation, and to promote their use as a functional food ingredient.

## 2. Materials and Methods

### 2.1. Materials

The water extract of jujube seeds (ZW) was provided by Serom Bio Co. (Gunpo, Korea). Jujube seeds were identified and authenticated by Prof. Kwang-Soon Shin of Kyonggi University, Suwon, Republic of Korea. A voucher specimen (KUCHS-220301) was deposited at the College of Health Sciences, Korea University, Seoul, Republic of Korea. Bicuculline (BIC), picrotoxin (PIX), and flumazenil (FMZ) were purchased from Sigma-Aldrich (St. Louis, MO, USA). Pentobarbital sodium and BDZ were obtained from Hanlim Pharmaceuticals Co., Ltd. (Yongin, Republic of Korea) and Pfizer (Seoul, Republic of Korea), respectively. Spezyme LT 300 (α-amylase) and Betalase 1500 EL (β-amylase) were acquired from Vision BioChem (Seongnam, Republic of Korea).

### 2.2. Ethanol Precipitation and Enzymatic Hydrolysis of ZW

After dissolving 100 g of ZW in 1 L of distilled water, 100% ethanol (four times, *v*/*v* = 1:4) was added and left at 4 °C. Following overnight incubation, the supernatant was obtained by centrifugation (4000 rpm, 10 min, 4 °C), freeze-dried, and used as alcohol-treated ZW (ZW-AT).

Ten grams of ZW were dissolved in 100 mL of distilled water (*w*/*v* = 1:10) and the pH was adjusted to 5.5. Next, 1% each of Spezyme LT 300 and Betalase 1500 EL were added and allowed to react at 60 °C for 12 h. Thereafter, the enzymes were inactivated by heat treatment (100 °C, 15 min), and the supernatant was collected by centrifugation (4000 rpm, 10 min, 4 °C) and lyophilized to obtain an enzyme hydrolysate of jujube seed extract (ZW-EH).

### 2.3. Fermentation by Lactobacillus brevis L32

ZW was hydrolyzed for 12 h using Spezyme LT 300 and Betalase 1500 EL. The hydrolyzed solution was adjusted to pH 6.0, autoclaved, and inoculated with a 4% pre-culture of *L. brevis*. The fermented product of jujube seed extract (ZW-FM) was obtained by fermentation at 30 °C for 48 h. Pre-culture of *L. brevis* L32 was obtained by culturing it at 30 °C for 24 h using de Man, Rogosa, and Sharpe medium.

### 2.4. Analysis of Glutamate and GABA Content

Amino acid analysis was used to quantify GABA and glutamate levels by high-performance liquid chromatography. Briefly, the fermentation broth was filtered through a 0.45 μm PVDF membrane. The filtered sample was derivatized with 6-aminoquioly-N-hydroxysuccinimidyl carbonate, and the derivatives were separated using an AccQ-Tag column (3.9 × 150 mm, Waters, Milford, MA, USA) [17]. GABA and glutamic acid contents were calculated using a standard curve prepared using GABA and glutamic acid (Sigma, St. Louis, MO, USA).

### 2.5. Animals

Five-week-old male Institute of Cancer Research (ICR) mice weighing approximately 25–27 g were procured from Orient Bio (Seongnam, Republic of Korea). The experiments were initiated after approval from the Korea University Animal Experiment Ethics Committee (approval number KUIACUC-2021–0100, dated 25 May 2022). The mice were bred in an animal house with automatically controlled light (12 h light/dark cycle), humidity (55.5%), and temperature (22 °C), with free access to pelleted diet (Altromin 1310, Lage, Germany) and water. All animals were used in the experiments after at least a week of acclimatization and were randomly assigned to groups based on the average weight of each group.

### 2.6. Pentobarbital-Induced Sleep Tests in Mice

Sleep induction experiments were conducted using pentobarbital. For this, all the mice were fasted for 24 h. Animals were randomly divided into 13 groups (seven mice/group); the normal (NOR) group was orally administered 0.9% physiological saline, and the positive control group was administered benzodiazepine (BDZ, 0.2 mg/kg) [18]. The extracts were dissolved in saline and orally administered to the animals (ZW, 150 and 200 mg/kg; ZW-AT, 150 and 200 mg/kg; ZW-EH, 150 and 200 mg/kg; ZW-FM, 50, 80, 100, 150, and 200 mg/kg). After 40 min, pentobarbital (42 mg/kg) was injected intraperitoneally (i.p.) to induce sleep, following which sleep latency and sleep duration time were recorded [18].

The sleep latency and sleep duration time of jujube extracts were also measured in a caffeine-induced insomnia model. The experimental groups were a normal group (NOR; treated with saline), control group (CON; treated with 40 mg/kg of caffeine), positive control group (BDZ; treated with caffeine and 0.2 mg/kg of benzodiazepine), and ZW-FM groups (treated with caffeine and 80, 100, and 150 mg/kg of ZW-FM, respectively). After 40 min of oral administration, pentobarbital (42 mg/kg, i.p.) was administered to measure sleep latency and sleep duration.

### 2.7. Evaluation of Sleep-Inducing Activity

After inhalation anesthesia with isoflurane, Sprague Dawley rats (250 g, male) were fixed on a stereotaxic device, and electroencephalogram electrodes were inserted according to the Paxinos and Watson anatomy. After surgery, the rats were allowed to recover for one week, following which an electroencephalography (EEG) transmitter was attached. Rats were randomly divided into four groups (six rats/group): normal group (NOR; treated with saline), positive control group (BDZ; treated with 0.2 mg/kg of benzodiazepine), and ZW-FM groups (treated with 100 and 150 mg/kg of ZW-FM). EEG was performed for 6 days at 15 mm/s for 7 h from 10:00 am to 17:00 h, based on the time of oral administration of the extracts. Sleep structure analysis was performed using the fast Fourier transform algorithm and the ecgAUTO3 program (Ver, 3.3, EMKA Technologies, Paris, France). To evaluate the sleep induction activity of the extracts, following electroencephalogram measurements, total sleep and sleep quality were evaluated by analyzing EEG potentials through baseline, control, and experimental recordings. Thirty minutes after extract administration, the time of rapid eye movement (REM), awake, δ, and θ waves were measured to determine the time of REM and non-REM sleep to assess improvement in sleep quality [19].

The EEG was also analyzed for 4 days in the caffeine-induced insomnia model. The experimental groups were a normal group (NOR; treated with saline), control group (CON; treated with 40 mg/kg of caffeine), positive control group (BDZ; treated with caffeine and 0.2 mg/kg of benzodiazepine), and ZW-FM group (treated with caffeine and 150 mg/kg of ZW-FM).

### 2.8. Analysis of the Receptor Binding Mode Using an Antagonist in Mice

Animals were randomly divided into eight groups (seven mice/group): normal group (NOR, i.p. injection of edible oil and 15 min later, orally administered saline), ZW-FM group (i.p. injection of edible oil and 15 min later, orally administered 150 mg/kg of ZW-FM), PIX groups (i.p. injection of PIX and 15 min later, orally administered saline or 150 mg/kg of ZW-FM), BIC groups (i.p. injection of BIC and 15 min later, orally administered saline or 150 mg/kg of ZW-FM), and FMZ groups (i.p. injection of FMZ and 15 min later, orally administered saline or 150 mg/kg of ZW-FM). PIX, BIC, and FMZ were dissolved at a concentration of 4, 6, and 10 mg/kg in edible oil, respectively. Sleep latency and sleep duration time were measured after administration of pentobarbital (42 mg/kg, i.p.) 40 min after the oral administration of the saline or ZW-FM extract [20].

### 2.9. Statistical Analysis

Data are expressed as mean ± standard error of the mean (SEM). Statistical analyses were performed using the Statistical Package for the Social Sciences (version 25.0; SPSS Inc., Chicago, IL, USA). Data were analyzed using ANOVA, and the significance of differences between samples was assessed using Tukey’s test at 95% significance.

## 3. Results

### 3.1. Effects of ZW, ZW-AT, and ZW-EH on Sleep Latency Time and Duration in Pentobarbital-Treated Mice

**Figure 1:**
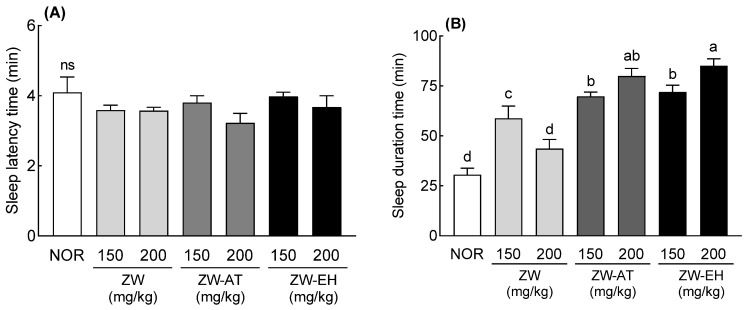
Effects of ZW, ZW-AT, and ZW-EH on sleep latency (**A**) and duration time (**B**) in pentobarbital-treated mice at the indicated concentrations. Data are presented as means ± standard error of the mean (*n* = 7). Different letters (a–d) indicate significant differences at *p* < 0.05 using Tukey’s test. ns, no significance compared to the NOR group; NOR, normal group; ZW, water extract of jujube seed; ZW-AT, supernatant of alcohol treated ZW; ZW-EH, enzymatic hydrolysate of ZW.

Changes in sleep latency and sleep duration time were measured in the pentobarbital-induced sleep mouse model following treatment of ZW with alcohol (ZW-AT) or enzymatic hydrolysis (ZW-EH). Sleep latency time tended to decrease in the groups administered 200 mg/kg (ZW, ZW-AT, and ZW-EH) compared to the NOR group, but the difference was not significant (Figure 1A). Sleep duration time was significantly increased in the ZW 150 mg/kg group compared to that in the NOR group (*p* < 0.001); however, when 200 mg/kg was administered, the sleep duration time decreased, and there was no significant difference from the NOR group (Figure 1B). With an increase in the dose of ZW-AT or ZW-EH, the sleep duration increased significantly compared to that in the NOR group (Figure 1B). In addition, ZW-AT and ZW-EH showed significantly increased sleep time compared to the same concentration of ZW. There was no significant difference in the sleep durations between the ZW-AT and ZW-EH groups, but the sleep time tended to increase in the ZW-EH group compared to the ZW-AT. The starch content of ZW-AT and ZW-EH was lower than that of ZW (Figure S1). Carbohydrates, especially starch, contained in ZW were eliminated by alcohol or amylase treatment; therefore, it is likely that sleep activity increased with an increase in the dose administered. Following an hour’s treatment of ZW with the starch-degrading enzymes α- and β-amylase, it was seen that the starch content decreased rapidly along with a rapid increase in the content of reducing sugar (Figure S2). There was no significant difference in the decrease in starch content following enzyme treatment for over 1 h; however, the reducing sugar content gradually increased, with the highest reducing sugar content being noted in the 12 h treatment. The starch hydrolase treatment proceeded for 12 h and the resulting extract was used as the fermentation sample for GABA conversion.

### 3.2. Changes in GABA Content, pH, Turbidity, and Reducing Sugar during Fermentation by L. brevis L-32 after Enzymatic Decomposition of Water Extract from Jujube Seed

**Figure 2:**
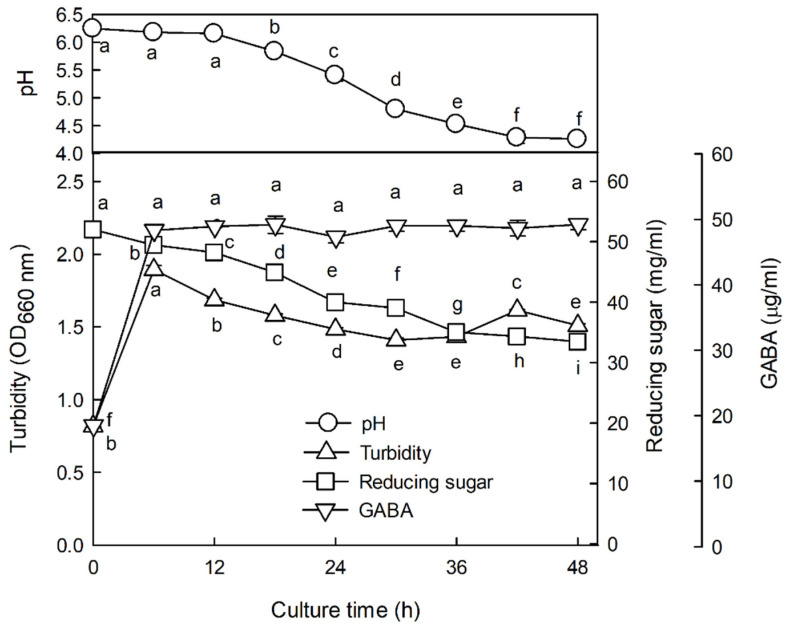
Changes in pH, reducing sugar, and GABA contents during fermentation of jujube seed extract by *Lactobacillus brevis* L32 after enzymatic hydrolysis. Data are presented as means ± standard error of the mean (*n* = 3). Different letters (a–i) indicate significant differences at *p* < 0.05 using Tukey’s test. GABA, γ-aminobutyric acid.

Figure 2 shows the changes in pH, reduced sugar, and GABA content during the fermentation of the jujube seed extract. As seen from the figure, at the beginning of fermentation the pH was 6.27, and as fermentation progressed, the pH gradually decreased; after 48 h, the pH was 4.26. The content of reducing sugar also showed a tendency to gradually decrease as the fermentation proceeded (33.46 at 48 h vs. 52.05 mg/mL at the beginning of fermentation), with the lowest concentration at 36 h. Turbidity, indicative of the extent of cell growth, increased rapidly until 6 h of fermentation and slowly decreased thereafter. During the fermentation process of jujube seed extract, the content of GABA increased rapidly to 48.34 μg/mL until 6 h of fermentation, subsequently reaching 47.31–49.21 μg/mL. The highest concentration of GABA was 49.21 μg/mL after 18 h of fermentation, but there was no significant difference in its concentration after 6 h. Therefore, the 36 h fermented product with high GABA and the lowest reducing sugar content was used as ZW-FM, a fermented product with enhanced sleep activity.

### 3.3. Effects of ZW-FM on Sleep Latency and Sleep Duration in Pentobarbital-Treated Mice

**Figure 3:**
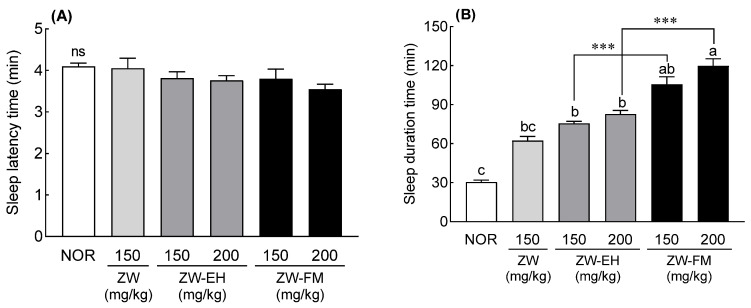
Effects of ZW, ZW-EH, and ZW-FM on sleep latency (**A**) and duration time (**B**) in pentobarbital-treated mice at the indicated concentrations. Data are presented as means ± standard error of the mean (*n* = 7). Different letters (a–c) indicate significant differences at *p* < 0.05 using Tukey’s test; *** *p* < 0.001 between ZW-EH and ZW-FM groups at the same dose based on one-way ANOVA followed by Tukey’s multiple comparison test. ns, not significant between the groups; NOR, normal group; ZW, water extract of jujube seed; ZW-EH, enzymatic hydrolysate of ZW; ZW-FM, fermented product of ZW-EH.

To evaluate the sleep-enhancing activity of ZW-FM and ZW-EH, sleep latency and duration were measured in a pentobarbital sleep-induced model. The ZW-EH and ZW-FM groups showed a tendency to decrease sleep latency when treated at a concentration of 200 mg/kg, but no significant difference was found as compared to the NOR group (Figure 3A). Sleep duration significantly increased by 1.21 times and 1.70 times in the ZW-EH and ZW-FM groups, respectively, compared to that in the ZW group at the concentration of 150 mg/kg (Figure 3B). In addition, at 200 mg/kg, the sleep time of the ZW-EH group was 82.76 ± 2.85 min (*p* < 0.001) and that of the ZW-FM group was 119.76 ± 5.55 min (*p* < 0.001), which was significantly higher than that of the NOR group (30.57 ± 1.37 min). At this dose, the fermentation product of ZW-FM resulted in a significantly longer sleep duration than ZW-EH (*p* < 0.001). The findings indicate that the fermentation of the jujube seed extract by *L. brevis* and enzymatic hydrolysis effectively increased the sleep-enhancing activity.

**Figure 4:**
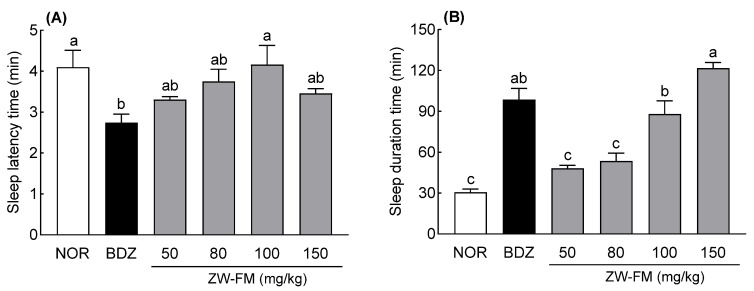
Effects of ZW-FM on sleep latency (**A**) and duration time (**B**) in pentobarbital-treated mice at the indicated concentrations. Data are presented as means ± standard error of the mean (*n* = 7). Different letters (a–c) indicate significant differences at *p* < 0.05 using Tukey’s test. NOR, normal group; BDZ, benzodiazepine, positive control group (0.2 mg/kg); ZW-FM, fermented product of ZW-EH; ZW-EH: enzymatic hydrolysate of jujube seed water extract (ZW).

Next, the sleep-enhancing activity was evaluated at 50–150 mg/kg ZW-FM. The positive control (BDZ) group showed a significant decrease in sleep latency compared to the NOR group (*p* < 0.05, Figure 4A). There was no significant difference in sleep latency between the ZW-FM (50–150 mg/kg) and NOR groups. In addition, in comparison to the NOR group, the sleep time significantly increased 3.22 times in the BDZ group (*p* < 0.001, Figure 4B), while the ZW-FM-administered groups (100 and 150 mg/kg) showed significantly increased sleep time compared to the NOR group (*p* < 0.001; *p* < 0.001, Figure 4B). This was particularly noted in the ZW-FM group (150 mg/kg), which showed a 3.98-fold increase in sleep time compared to that in the NOR group.

### 3.4. Effect of ZW-FM on REM and Non-REM Sleep

**Figure 5:**
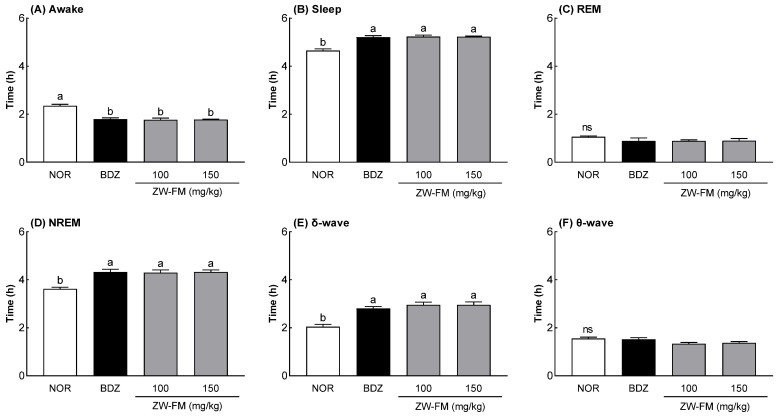
Effects of ZW-FM on rapid eye movement (REM) and non-REM sleep in rats at the indicated concentrations. Data are presented as means ± standard error of the mean (*n* = 6). Different letters (a,b) indicate significant differences at *p* < 0.05 using Tukey’s test. ns, not significant between the groups; NOR, normal group; BDZ, benzodiazepine (0.2 mg/kg); ZW-FM, fermented product of ZW-EH; ZW-EH, enzymatic hydrolysate of jujube seed water extract (ZW).

Having confirmed the sleep-enhancing activity of ZW-FM, EEG analysis was used to measure its effect on REM and non-REM sleep (Figure 5). When compared to the NOR group, there was a significant decrease in the awake time in the ZW-FM groups (100 and 150 mg/kg) (*p* < 0.001) along with a significant increase in the sleep time (*p* < 0.001). There was no significant difference in REM sleep time between the groups, but non-REM sleep time increased significantly in the BDZ and ZW-FM administrated groups compared to the NOR group. The results of measuring δ and θ waves, the brain waves constituting non-REM sleep, showed that in the ZW-FM groups, δ waves increased significantly more than in the NOR group, but there was no significant difference between the groups with regards to the θ waves. Sleep enhancement by administration of the fermented product of jujube extract was attributed to an increase in non-REM sleep time due to an increase in δ waves.

### 3.5. Effect of ZW-FM on REM and Non-REM Sleep

**Figure 6:**
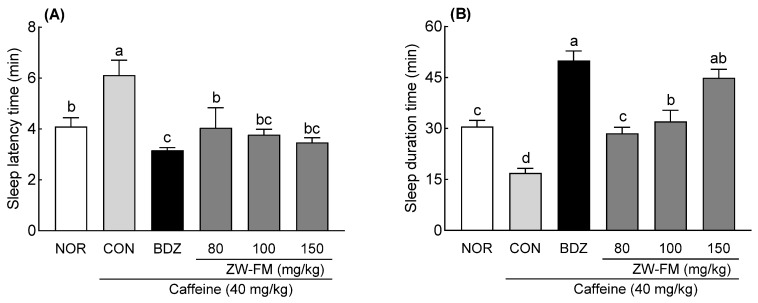
Effect of ZW-FM on sleep latency (**A**) and total sleep duration time (**B**) in caffeine-induced insomnia model at the indicated concentrations. Data are presented as means ± standard error of the mean (*n* = 7). Different letters (a–d) indicate significant differences at *p* < 0.05 using Tukey’s test. NOR, normal group; CON: caffeine (40 mg/kg) control group; BDZ, benzodiazepine (0.2 mg/kg)-treated group; ZW-FM, fermented product of ZW-EH-treated groups; ZW-EH: enzymatic hydrolysate of jujube seed water extract (ZW).

A pentobarbital-induced sleep test was conducted using a mouse model of caffeine-induced insomnia. In the CON group treated with caffeine alone, compared to that in the NOR group, sleep latency time significantly increased (*p* < 0.05, Figure 6A), while sleep duration significantly decreased (*p* < 0.01, Figure 6B), confirming that caffeine administration induced insomnia. The positive control group (BDZ) showed a significant decrease in sleep latency time compared to the CON group (*p* < 0.01). The sleep latency time in the CON group was 6.12 ± 0.59 min, and in comparison, there was a significant decrease in the ZW-FM groups (80, 100, and 150 mg/kg), with the sleep latency time being 4.81 ± 0.96 min (*p* < 0.05). 3.82 ± 0.40 min (*p* < 0.01), and 3.25 ± 0.20 min (*p* < 0.01), respectively; the decrease being dose dependent. In addition, sleep duration time was significantly increased in the BDZ group compared to the CON group (*p* < 0.001), and the ZW-FM groups (80, 100, and 150 mg/kg) had significantly increased sleep duration time compared to the CON group (16.93 ± 1.34 min), with 28.56 ± 1.80 min (*p* < 0.05), 32.08 ± 3.30 min (*p* < 0.01), and 44.90 ± 2.50 min (*p* < 0.001), respectively.

**Figure 7:**
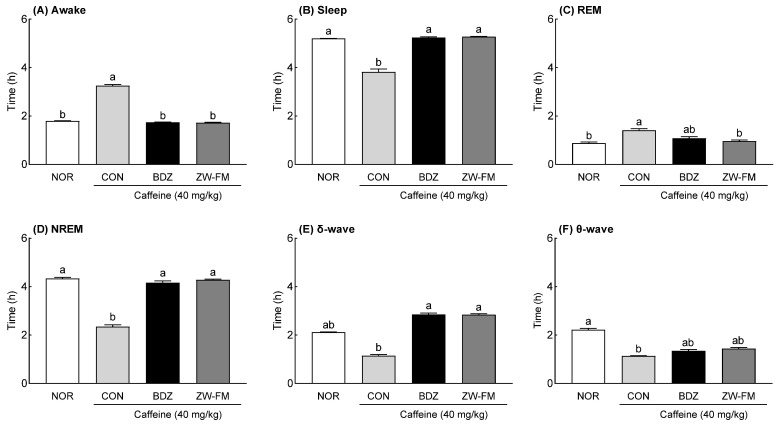
Effect of ZW-FM on REM and non-REM sleep in caffeine-induced insomnia model at the indicated concentration. Graphs indicate wake time (**A**), sleep time (**B**), REM (**C**), NREM (**D**), δ wave (**E**), and θ waves (**F**) in indicated groups. Data are presented as means ± standard error of the mean (*n* = 6). Different letters (a,b) indicate significant differences at *p* < 0.05 using Tukey’s test. NOR, normal group; CON, caffeine (40 mg/kg) control group; BDZ, benzodiazepine (0.2 mg/kg)-treated group; ZW-FM: fermented product of ZW-EH -treated group.

The EEG analysis showed that in the CON group, sleep (*p* < 0.001, Figure 7B), non-rapid eye movement (NREM) sleep (*p* < 0.001, Figure 7D), δ-wave (*p* < 0.001, Figure 7E), and θ-wave (*p* < 0.001, Figure 7F) times were significantly decreased compared to the NOR group, and awake (*p* < 0.001, Figure 7A) and REM sleep (*p* < 0.001, Figure 7C) times were significantly increased. In the insomnia model, the BDZ group showed a significant decrease in awake time (*p* < 0.001) and a significant increase in sleep time (*p* < 0.001) compared with the CON group. The ZW-FM group (150 mg/kg) showed similar results to the BDZ group. However, REM sleep time was 1.08 ± 0.07 h in the BDZ group (*p* < 0.05) and 0.96 ± 0.05 h in the ZW-FM group (*p* < 0.01); the decrease was more prominent in the ZW-FM group compared to that in the CON group (1.41 ± 0.06 h). With respect to the NREM sleep time, as compared to the CON group (2.34 ± 0.09 h), there was a significant increase in the BDZ (4.16 ± 0.08 h, *p* < 0.001) and ZW-FM (4.28 ± 0.03 h, *p* < 0.001) groups. In particular, ZW-FM (150 mg/kg) increased the δ-wave (*p* < 0.001) and θ-wave (*p* < 0.01) by 2.47 and 1.27 times, respectively, compared to the CON group. As ZW-FM, a fermented product of jujube seed extract, shows sleep-enhancing activity in an insomnia model, it may be effective in people with sleep disorders.

### 3.6. Effects of GABA_A_ Receptor Antagonists on the Sleep-Enhancing Activity of ZW-FM in Pentobarbital-Treated Mice

**Figure 8:**
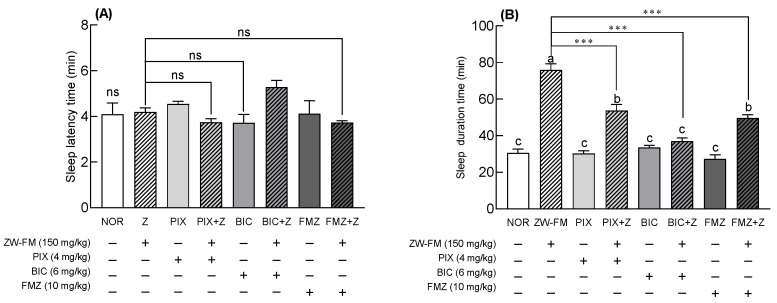
Effects of GABA_A_ receptor antagonists on sleep latency (**A**) and sleep duration (**B**) in mice treated with a hypnotic dose of pentobarbital. Mice were orally administered with ZW-FM (150 mg/kg). Data are presented as means ± standard error of the mean (*n* = 7). Different letters (a–c) indicate significant differences at *p* < 0.05 using Tukey’s test. *** *p* < 0.001 as compared between antagonist treatment and antagonist non-treatment groups based on one-way ANOVA followed by Tukey’s multiple comparison test. NOR, normal group; ZW-FM, fermented product of ZW-EH; PIX, picrotoxin (4 mg/kg); BIC, bicuculline (6 mg/kg); FMZ, flumazenil (10 mg/kg); ZW-EH, enzymatic hydrolysate of jujube seed water extract (ZW); GABA: γ-aminobutyric acid; ns, not significant between the groups.

Based on the pentobarbital-induced sleep test using GABA_A_ receptor antagonists, the sleep latency time showed no significant change compared to the NOR group after ZW-FM or antagonist (PIX, BIC, and FMZ) treatment (Figure 8A). The group treated with only antagonists PIX, BIC, and FMZ showed no significant effect on sleep time compared with the NOR group (Figure 8B). In contrast, PIX, BIC, and FMZ significantly reduced the increased sleep duration induced by ZW-FM treatment (*p* < 0.001). In particular, the sleep time of the group treated with BIC and ZW-FM (150 mg/kg) was 36.90 ± 1.84 min, significantly decreased compared to the ZW-FM group (75.93 ± 3.44 min) and similar to that of the NOR group (30.57 ± 2.12 min). These results suggest that the GABA_A_ receptor was involved in the sleep-enhancing activity of ZW-FM.

## 4. Discussion

GABA is a non-protein constituent amino acid that is widely distributed in nature, including in animals and plants. Various physiological effects of GABA, such as increased brain function, have been documented [14,21]. In general, the inhibition of GABA action can cause anxiety and convulsions, whereas increased action induces antidepressant, anticonvulsant, and sedative effects [22]. As the functionality of GABA is known, interest in it is increasing, not only as a medicine but also as a functional food ingredient. Owing to its low content, it is difficult to expect the physiological activity of GABA in the amount consumed as a natural food. Therefore, in this study, to increase the GABA content in the water extract of jujube seeds, fermentation was performed using *L. brevis* L32, and the sleep-enhancing activity of the fermented product was evaluated.

Jujube seeds contained 8.8% starch, and the aqueous extract also had starch. The sleep duration of the ZW was lower than that of 150 mg/kg when 200 mg/kg was administered (Figure 1B). However, when ZW was treated with alcohol- or starch-degrading enzymes, sleep duration increased as the concentration increased (Figure 1B). These results are likely due to the presence of starch in the aqueous extract. Amylose, a component of starch, forms a helical structure through 𝛼-D-(1–4) glycosidic bonds. These helical structures can form complexes with polar and non-polar compounds [23]. Therefore, it is speculated that the sleep duration time was reduced at high concentrations because the starch contained in the water extract formed a complex with sleep-enhancing substances. However, as the starch was removed by alcohol treatment or starch-degrading enzyme treatment, the sleep duration time increased as the dose increased. A previous study reported that GABA contained in jujube fruit showed a sleep improvement effect [13]. However, the content of GABA in common plants is insufficient to expect pharmacological action. For this reason, studies to increase GABA levels have been actively conducted. In particular, microbial fermentation is one of the most promising methods for obtaining GABA, and *L. brevis* is known as a strain with high GABA production ability [24]. A recent study revealed that GABA-fermented milk obtained by fermenting milk with *L. brevis* prolonged sleep time and shortened sleep latency in mice [25]. Another study showed that the biotransformation of monosodium glutamate to GABA by *L. brevis* produced GABA with a conversion rate of 65.6%. Additionally, when mice were given this biomodified GABA, their sleep duration increased [26]. In our study, ZW-FM (fermented product of jujube seed water extract) increased sleep duration time by 1.39–1.44 times compared to the ZW-EH treated groups at the same concentration (Figure 3). The increase in GABA content by fermentation showed a higher increase in sleep time compared to that before fermentation (Figure 2).

Sleep can be divided into two types: REM and NREM [27]. NREM sleep can be divided into three stages (N1 sleep, stage N1; N2 sleep, stage N2; and N3 sleep, stage N3) according to sleep depth. Higher levels of deep sleep require stronger stimuli to switch to wakefulness. NREM sleep starts with the N1 or N2 stages, which have a relatively low depth, and further progresses to the N3 stage (slow-wave sleep), characterized by high-amplitude δ waves [27,28]. A decrease in the slow-wave ratio has been reported in insomnia [29], and an increase in this wave improves sleep disorders and affects sleep quality [29]. An increase in sleep time by ZW-FM as seen by an increased NREM can be attributed to an increase in δ waves (Figure 5). Slow-wave sleep by δ waves indicates the deepest sleep, and since ZW-FM administration increased sleep time and quality, the improvement can be due to an increase in slow-wave sleep.

In the caffeine-induced insomnia model, ZW-FM administration also increased sleep duration (Figure 6) due to an increase in non-REM sleep time (Figure 7). In this model, administration of ZW-FM significantly increased δ- and θ-waves as compared to that in the CON group. This seems to be different from the EEG changes observed in the normal model induced by caffeine administration. Caffeine, belonging to the methylxanthine class, enhances awakeness by blocking adenosine receptors and increasing excitatory neurotransmitter release [30]. Caffeine induces various sleep disturbances in humans and mice, including decreased total sleep time, prolonged sleep-onset latency, and increased wakefulness [31].

The sleep-enhancing activity of jujube seems to be due to the sedative-sleep effect influenced by the expression levels of GABA_A_ α1, α5, and β2 by jujuboside, reported to be an active compound [6]. Jujuboside B and jujubogenin, which are saponin metabolites, interact with GABA_A_ receptors and act as sedatives [32]. A possible mechanism for the sleep-enhancing activity of ZW-FM is through the GABA_A_ receptor in the brain tissue. Treatment with GABA_A_ receptor antagonists PIX, BIC, and FMZ reduced the sleep-enhancing activity of ZW-FM (Figure 8). GABA_A_ receptor is the major receptor for sleep function and is composed of 19 different GABA_A_ subunits (α1–6, β1–3, γ1–3, δ, ε, θ, π, and ρ1–3) [33]. Scientific evidence from various models of GABA_A_ receptors indicates a direct connection with sleep [16,34]. Various agonists and antagonists have been identified that act on the GABA_A_ receptor. BIC is an antagonist binding to the GABA site of the GABA_A_ receptor, PIX is an antagonist that binds to the picrotoxin site located inside the chloride channel of the GABA_A_ receptor, and FMZ is known as an antagonist that binds to the benzodiazepine site of the GABA_A_ receptor [35,36]. Among the GABA_A_ receptor antagonists, in particular, BIC reduced the sleep-enhancing activity of ZW-FM to a level similar to that of the NOR group, which is speculated to be due to an increased content of GABA and glutamate following fermentation. Therefore, ZW-FM binds most effectively to the GABA_A_ receptor, and when combined with previous results, it can be stated that ZW-FM exhibits sleep-enhancing activity via GABAergic action.

## 5. Conclusions

In this study, the water extract of jujube seeds from which starch was removed using starch-degrading enzymes was fermented with *L. brevis* to obtain a fermented product (ZW-FM) with high GABA content. ZW-FM increased total sleep time in the rodent model and improved sleep quality by increasing δ-waves, especially during NREM sleep time. ZW-FM also attenuated arousal in a caffeine-induced insomnia model. Studies have also demonstrated that sleep is inhibited by GABA_A_ receptor antagonists picrotoxin, bicuculline, and flumazenil. Thus, ZW-FM has been shown to enhance sleep activity in rodent models through GABAergic-mediated behavior. In conclusion, this study provides evidence supporting the potential usefulness of ZW-FM with increased GABA content in promoting sleep.

## Data Availability

The data that support the findings of this study are available from the corresponding author upon reasonable request.

## Supplementary Materials

The following supporting information can be downloaded at: https://www.mdpi.com/article/10.3390/foods12152864/s1, Figure S1. Changes in starch and reducing sugar content of jujube seed extract after enzymatic or alcohol treatment. Data are presented as means ± standard error of the mean (*n* = 3). *** *p* < 0.001 vs. ZW group. ZW, water extract of jujube seed; ZW-AT, supernatant of alcohol treated ZW; ZW-EH, enzymatic hydrolysate of ZW, Figure S2. Changes in starch and reducing sugar content of jujube seed extract after α- and β-amylase treatment. Data are presented as means ± standard error of the mean (*n* = 3). Different letters (a–d) indicate significant differences at *p* < 0.05 using Tukey’s test.
