# Supplementary material for: Sleep-Enhancing Effect of Water Extract from Jujube (Zizyphus jujuba Mill.) Seeds Fermented by Lactobacillus brevis L32
Source: Foods. 2023 Jul 27;12(15):2864. doi: 10.3390/foods12152864 (PMC10417159; doi:10.3390/foods12152864)
Supplement: Supplementary file 1 [file foods-12-02864-s001.zip › Jujube_Supplementary_Figure_S1_Revision.pdf]

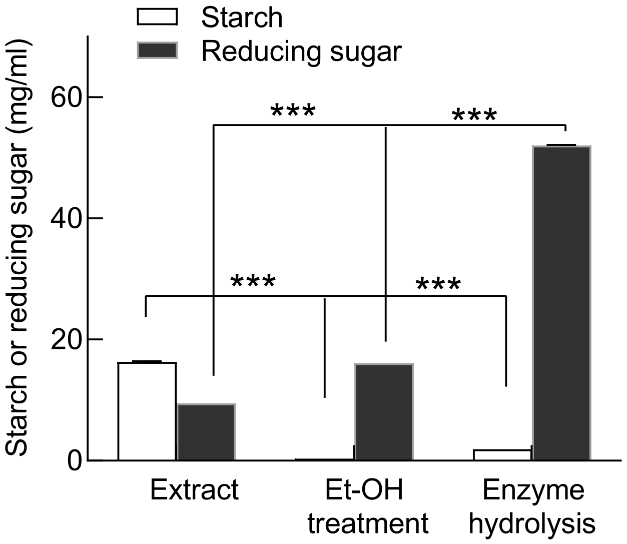

Figure S1. Changes in starch and reducing sugar content of jujube seed extract after enzymatic or alcohol treatment. Data are presented as means ± standard error of the mean (n=3). ***$p<0.001$ vs. ZW group. ZW: water extract of jujube seed, ZW-AT: supernatant of alcohol treated ZW, ZW-EH: enzymatic hydrolysate of ZW.
