# Supplementary material for: Sleep-Enhancing Effect of Water Extract from Jujube (Zizyphus jujuba Mill.) Seeds Fermented by Lactobacillus brevis L32
Source: Foods. 2023 Jul 27;12(15):2864. doi: 10.3390/foods12152864 (PMC10417159; doi:10.3390/foods12152864)
Supplement: Supplementary file 1 [file foods-12-02864-s001.zip › Jujube_Supplementary_Figure_S2_Revision.pdf]

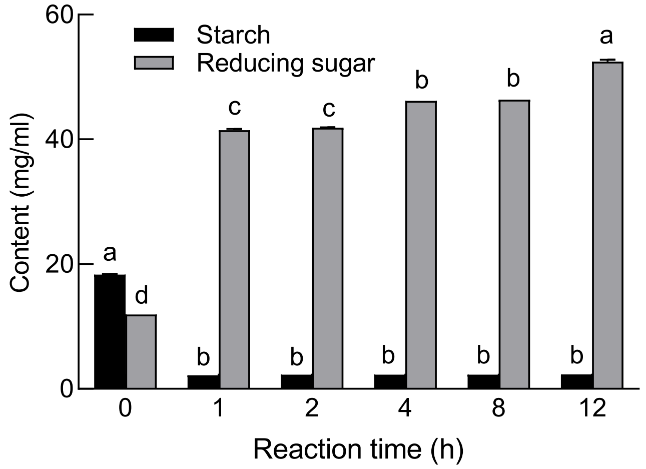


**Figure S2**. Changes in starch and reducing sugar content of jujube seed extract after $\alpha$- and β-amylase treatment. Data are presented as means ± standard error of the mean (n=3). Different letters (a-d) indicate significant differences at $p<0.05$ using Tukey's test.
